# Lung Abscess and Empyema Caused by Panton‐Valentine Leukocidin‐Positive Methicillin‐Resistant *Staphylococcus aureus* ψUSA300 Strain in a Previously Healthy Adult

**DOI:** 10.1002/rcr2.70772

**Published:** 2026-10-28

**Authors:** Asako Yanagisawa, Yuri Emori, Hodaka Serizawa, Rikako Nakamura, Daisuke Kishikawa, Hiroshi Kojima, Hidehito Matsui, Hidefumi Shimizu

**Affiliations:** ^1^ Department of Respirology Japan Community Healthcare Organization (JCHO) Tokyo Shinjuku Medical Center Tokyo Japan; ^2^ Ōmura Satoshi Memorial Institute, Kitasato University, Minato‐Ku Tokyo Japan

**Keywords:** ψUSA300, empyema, MRSA, Panton‐Valentine leukocidin (PVL)

## Abstract

PVL‐positive ψUSA300 CA‐MRSA is increasingly identified in Japan and is primarily associated with skin and soft tissue infections; respiratory infections caused by this clone remain rare. We report a 49‐year‐old healthy woman with lung abscess and empyema caused by PVL‐positive ψUSA300, successfully treated with vancomycin and linezolid. This case expands the known clinical spectrum of ψUSA300 and underscores the need for CA‐MRSA consideration in otherwise healthy patients with severe pulmonary infections.

## Graphical abstract

ψUSA300 CA‐MRSA is an emerging pathogen in Japan, rarely reported as a cause of respiratory infection. We present a previously healthy woman who developed a severe lung abscess and empyema caused by PVL‐positive ψUSA300. Early anti‐MRSA therapy led to full recovery, underscoring the need for CA‐MRSA awareness in patients with lung abscess or empyema.

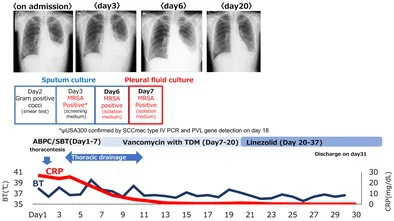

## 1 Introduction

Panton–Valentine leukocidin (PVL)‐positive community‐acquired methicillin‐resistant *Staphylococcus aureus* (CA‐MRSA) is associated with severe skin and soft tissue infections and occasionally causes invasive pulmonary infections. In Japan, the ψUSA300 clone has increasingly been identified among PVL‐positive CA‐MRSA isolates, particularly those associated with skin and soft tissue infections [1]; however, reports of respiratory infections caused by this strain remain rare [2]. We report a case of PVL‐positive ψUSA300 lung abscess and empyema in a previously healthy woman.

## 2 Case Report

A 49‐year‐old female homemaker developed sore throat followed by left‐sided chest pain 1 week later and was referred to our hospital. She frequently travels between Japan and New Zealand but had no overseas travel within 3 months of symptom onset. She had no significant past medical history, took no regular medications, and was a former smoker (2.5 pack‐years). She lives with her husband and one child.

On admission, the patient was alert and oriented. Her vital signs were: body temperature 38.6°C, blood pressure 120/74 mmHg and pulse rate 101 bpm. Oxygen saturation was 99% on room air. Her respiratory rate was 22 breaths/min. Breath sounds were diminished in the left lung field, with no cardiac murmurs, lower extremity edema or skin lesions such as wounds, abscesses or cellulitis. Laboratory tests on admission revealed leukocytosis (white blood cell count: 16,740/μL) and a markedly elevated C‐reactive protein level (24.37 mg/dL). Procalcitonin was mildly elevated at 0.59 ng/mL. Urinary antigen tests for *Legionella pneumophila* and *Streptococcus pneumoniae* , as well as rapid antigen tests for influenza virus and SARS‐CoV‐2, were all negative. (Table 1).

**TABLE 1:** Admission laboratory findings.

Category	Test	Ref. range	Unit	On admission
CBC	WBC	3.4–8.9	×10^3^/μL	16.7
	Hemoglobin	10.2–14.9	g/dL	11.5
	Hematocrit	33.5–46.1	%	34.5
	Platelet count	155–393	×10^3^/μL	185
Differential	Neutrophils	36.0–69.5	%	81.6
	Lymphocytes	20.8–52.7	%	4.1
	Monocytes	4.5–10.6	%	14.1
	Eosinophils	0.5–8.8	%	0.0
	Basophils	0.2–1.7	%	0.2
Biochemistry	Total Protein	6.7–8.3	g/dL	6.8
	Albumin	3.8–5.3	g/dL	3.1
	AST	10–40	U/L	11
	ALT	5–45	U/L	20
	LDH	100–225	U/L	157
	ALP	28–113	U/L	61
	Total Bilirubin	0.2–1.2	mg/dL	0.7
	BUN	7–22	mg/dL	10.7
	Creatinine	0.45–0.85	mg/dL	0.61
	Sodium (Na)	137–147	mEq/L	134
	Potassium (K)	3.5–5	mEq/L	3.4
	Chloride (Cl)	98–108	mEq/L	98
	CRP	0–0.299	mg/dL	24.37
	HbA1c	5.7–6.2	%	5.5
	PT (INR)	0.9–1.1	—	1.19
	APTT	24–39	s	43.9
	D‐dimer	∼1	μg/mL	2.97
Antigen tests	Legionella			Negative
	*Streptococcus pneumoniae*			Negative
	Influenza virus			Negative
	SARS‐CoV‐2			Negative
Pleural fluid analysis findings				
Appearance	Yellowish and slightly turbid			
pH				7.0
Cell count			/μL	26,075
Neutrophil			%	81.8
Protein			g/dL	5.2
LDH			U/L	637
Glucose			mg/dL	100
CEA			ng/mL	< 1.7
Adenosine deaminase			U/L	31.9

Abbreviations: ALP, alkaline phosphatase; ALT, alanine aminotransferase; APTT, activated partial thromboplastin time; AST, aspartate aminotransferase; BUN, blood urea nitrogen; CBC, complete blood count; CEA, carcinoembryonic antigen; CRP, C‐reactive protein; INR, international normalised ratio; LDH, lactate dehydrogenase; PT, prothrombin time; WBC, white blood cell count.

Chest radiography revealed infiltrative shadows in the left lower lung field with pleural effusion (Figure 1A). Chest computed tomography further demonstrated consolidation in the dorsal segment of the left lower lobe, accompanied by abscess formation and loculated pleural effusion (Figure 1B). Given the small volume of pleural effusion, only diagnostic thoracentesis was performed. Pleural fluid analysis revealed exudative effusion consistent with a complicated parapneumonic effusion (Table 1). Blood cultures drawn on admission yielded no growth.

**FIGURE 1:**
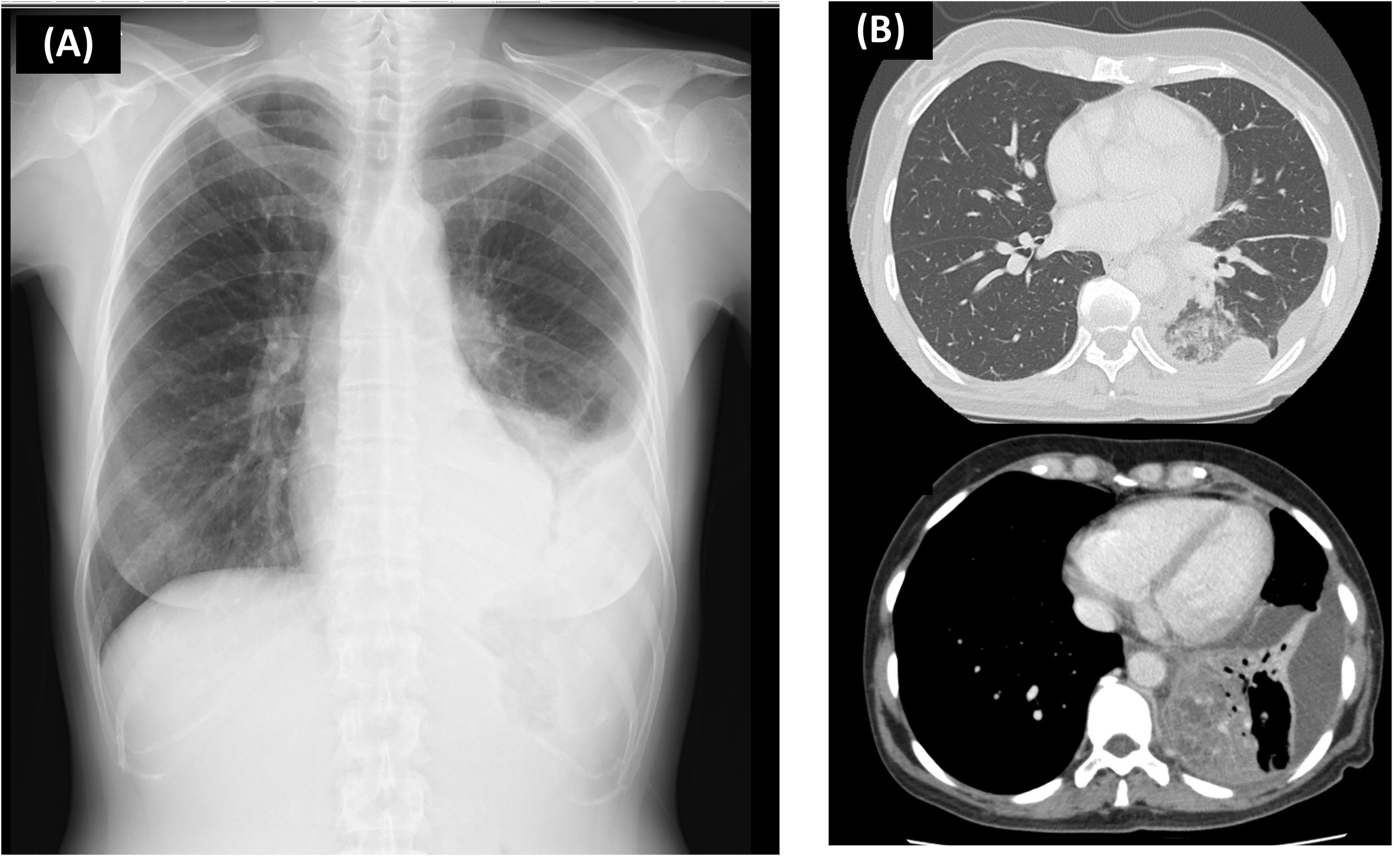
(A) Chest radiograph showing infiltrative shadow in the left lower lung field with pleural effusion. (B) Chest CT demonstrating consolidation with loculated pleural effusion and a low‐density area suggestive of abscess formation in the left lower lobe.

The patient was diagnosed with lung abscess and complicated parapneumonic effusion. Empirical treatment with sulbactam/ampicillin (SBT/ABPC) was initiated on Day 1 (Figure 2). Sputum Gram staining on Day 2 revealed Gram‐positive cocci. On Day 3, worsening pleural effusion required continuous thoracic drainage, and a sputum screening culture was presumptively positive for MRSA. However, because inflammatory markers and clinical symptoms had shown initial improvement under SBT/ABPC therapy, the clinical significance of this isolate was initially considered uncertain. Definitive isolation from the same specimen was confirmed on Day 6, and MRSA was also isolated from pleural fluid on Day 7. Consequently, therapy was switched to vancomycin (VCM; 15 mg/kg q12h) with therapeutic drug monitoring to target the MRSA. Following the initiation of VCM, inflammatory markers and chest radiographic findings improved rapidly. The thoracic drain was removed on Day 10. On Day 20, VCM‐induced neutropenia (1170/μL) prompted a switch to oral linezolid (LZD; 600 mg twice daily). Because MRSA pneumonia occurred in a young patient without underlying disease, CA‐MRSA was suspected and molecular analysis was performed. The isolate was confirmed to be Panton‐Valentine leukocidin (PVL)‐positive by PCR and was characterised as SCCmec Type IV, ACME Type I, and sequence type ST8 (clonal complex 8) by multiplex PCR and multilocus sequence typing (MLST). MLST was performed by sequencing seven housekeeping genes and comparing the resulting allelic profile with the PubMLST database. These findings—PVL positivity, SCCmec Type IV, ACME Type I and ST8 (CC8)—met the defining criteria for the ψUSA300 clone. The patient was discharged on Day 31. Despite a declining platelet count, oral LZD was tolerated and continued until Day 37. No recurrence or secondary transmission has occurred over a 2‐month follow‐up period. The patient expressed concern regarding possible transmission to family members and was relieved after receiving education regarding infection control measures and observing no secondary household cases during follow‐up.

**FIGURE 2:**
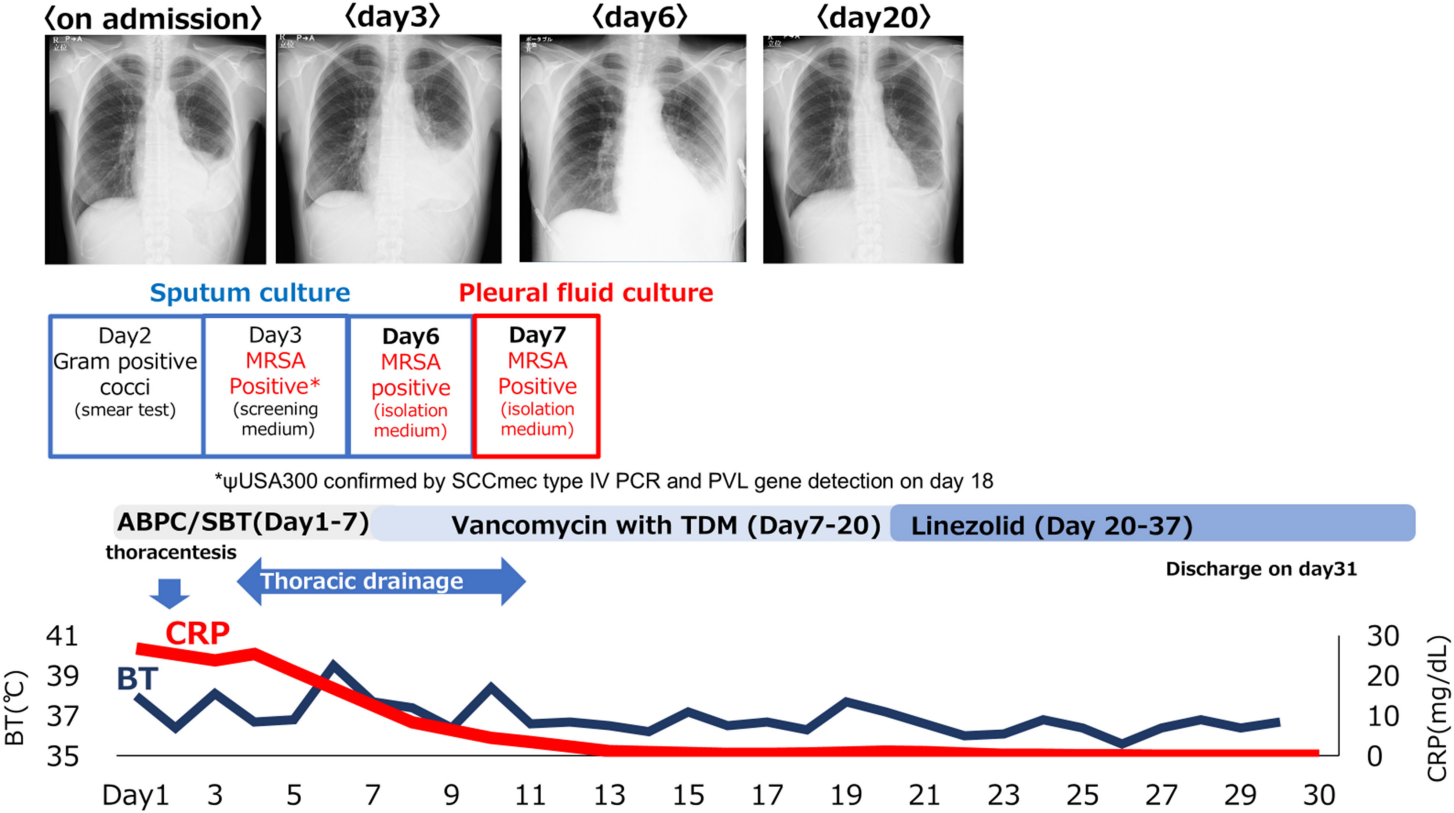
Clinical course showing the timing of antimicrobial therapy, thoracic drainage and key clinical events from hospital admission to discharge. BT, body temperature; CRP, C‐reactive protein; LZD, linezolid; PVL, Panton–Valentine leukocidin; SBT/ABPC, sulbactam/ampicillin; TDM, therapeutic drug monitoring; VCM, vancomycin.

## 3 Discussion

This case demonstrates that the PVL‐positive ψUSA300 clone can cause lung abscess and empyema despite a mild clinical onset. Although transient improvement in inflammatory markers initially obscured the pathogenic role of MRSA, timely diagnostic revision and targeted antimicrobial therapy resulted in complete resolution.

Initial improvement in inflammatory markers following β‐lactam therapy led clinicians to underestimate the pathogenic significance of MRSA recovered from respiratory specimens. However, the subsequent isolation of MRSA from pleural fluid and the favourable response to anti‐MRSA therapy supported its role as the causative pathogen. PVL‐positive MRSA pneumonia has been reported in association with severe influenza infection [3]. However, the preceding infection in this patient was mild and uncomplicated but may have facilitated MRSA colonisation and invasion.

The ψUSA300 clone has increasingly been identified among PVL‐positive CA‐MRSA isolates in Japan and is strongly associated with SSTIs [4]. To our knowledge, only one fatal case of ψUSA300 pneumonia has been reported; unlike that case involving bacteremia, our patient had no bloodstream infection and recovered completely [2]. Therefore, the pulmonary virulence of this clone remains poorly characterised. The present case demonstrates that ψUSA300 can cause severe lung abscess and empyema in an otherwise healthy individual; however, further case accumulation is required to clarify the clinical spectrum and severity of respiratory infections caused by this lineage.

Nasal swab cultures were not obtained from the patient or household members at the time of diagnosis, and the precise route of transmission could therefore not be determined. Given the absence of recent overseas travel and the increasing prevalence of the ψUSA300 clone in the Japanese community, domestic community‐acquired transmission is considered the most likely route. This lack of microbiological investigation represents a limitation of the present case. Future studies incorporating systematic screening of household members may help clarify the transmission dynamics of this clone.

Although respiratory infections caused by this clone remain rare, clinicians should maintain a high index of suspicion for CA‐MRSA in patients with lung abscess or empyema, even without comorbidities. Early anti‐MRSA therapy and source control are essential. Furthermore, because household contamination and colonised contacts contribute to CA‐MRSA transmission, guidance on household hygiene, environmental decontamination and targeted decolonisation when indicated may reduce recurrence and transmission risk [5]. Clear communication about MRSA also helps alleviate patient anxiety following diagnosis. This case highlights that ψUSA300 can cause severe lung abscess and empyema even in previously healthy individuals.

## Author Contributions

A.Y. drafted the manuscript. R.N., D.K., H.K. and H.S. were directly involved in the clinical management of the patient. H.M. performed the microbiological and molecular analyses. All authors critically revised the manuscript and approved the final version.

## Funding

The authors have nothing to report.

## Consent

The authors declare that written informed consent was obtained for the publication of this manuscript and accompanying images and attest that the form used to obtain consent from the patient complies with the Journal requirements as outlined in the author guidelines.

## Conflicts of Interest

The authors declare no conflicts of interest.

## Data Availability

Data sharing not applicable to this article as no datasets were generated or analysed during the current study.
